# Disruption of carbon for nutrient exchange between potato and arbuscular mycorrhizal fungi enhanced cyst nematode fitness and host pest tolerance

**DOI:** 10.1111/nph.17958

**Published:** 2022-02-02

**Authors:** Christopher A. Bell, Emily Magkourilou, P. E. Urwin, Katie J. Field

**Affiliations:** ^1^ Faculty of Biological Sciences School of Biology University of Leeds Leeds LS2 9JT UK; ^2^ Plants, Photosynthesis and Soil School of Biosciences University of Sheffield Sheffield S10 2TN UK

**Keywords:** arbuscular mycorrhizal fungi (AMF), carbon for nutrient exchange, host pest tolerance, plant‐parasitic nematodes, symbiosis

## Abstract

- Plants simultaneously interact with a range of biotrophic symbionts, ranging from mutualists such as arbuscular mycorrhizal fungi (AMF), to parasites such as the potato cyst nematode (PCN). The exchange of mycorrhizal‐acquired nutrients for plant‐fixed carbon (C) is well studied; however, the impact of competing symbionts remains underexplored.
- In this study, we examined mycorrhizal nutrient and host resource allocation in potato with and without AMF and PCN using radioisotope tracing, whilst determining the consequences of such allocation.
- The presence of PCN disrupted C for nutrient exchange between plants and AMF, with plant C overwhelmingly obtained by the nematodes. Despite this, AMF maintained transfer of nutrients on PCN‐infected potato, ultimately losing out in their C for nutrient exchange with the host. Whilst PCN exploited the greater nutrient reserves to drive population growth on AMF–potato, the fungus imparted tolerance to allow the host to bear the parasitic burden.
- Our findings provide important insights into the belowground dynamics of plant–AMF symbioses, where simultaneous nutritional and nonnutritional benefits conferred by AMF to hosts and their parasites are seldom considered in plant community dynamics. Our findings suggest this may be a critical oversight, particularly in the consideration of C and nutrient flows in plant and soil communities.

## Introduction

Symbioses between plants and mycorrhizal fungi evolved at the dawn of plant terrestrialization and are widely thought to have enhanced the capabilities of early plants to scavenge and assimilate nutrients from the skeletal mineral soils of the Early Devonian (Field & Pressel, 2018). In return, early plants likely transferred photosynthetically fixed organic carbon (C) (Field & Pressel, 2018), as hexose sugars (Helber *et al*., 2011) and lipids (Luginbuehl *et al*., 2017). Today, more than 80% of land plants associate with arbuscular mycorrhizal fungi (AMF) of the Glomeromycotina (Brundrett & Tedersoo, 2018), which form intimate partnerships with host plant roots and rhizoids (Smith & Read, 2008). These associations can play a critical role in plant nutrition, facilitating host plant access to soil nutrients, such as nitrogen (N) and phosphorus (P), from otherwise inaccessible sources in the soil (Smith & Read, 2008; Sato *et al*., 2015). As such, there is great potential in promoting symbioses between AMF and crops in agricultural environments with a view to enhancing soil nutrient uptake in crops while reducing the need for excessive fertilizer application (Leake *et al*., 2011).

AMF rely on their host plant as their primary source of organic C, which can lead to significant demand on the host for resources (Smith & Read, 2008). The transfer of C to AMF by hosts may be regulated by the host’s perception of nutrient delivery from the AMF. In certain scenarios, mycorrhizal fungal partners that provide a larger amount of nutrients to the host plant are ‘rewarded’ with a greater quantity of plant‐fixed C in return (Kiers *et al*., 2011). However, reciprocity in the C‐for‐nutrient exchange between symbionts is highly context dependent (Walder *et al*., 2012). In some cases, plant hosts appear to discriminate between symbionts, allocating C preferentially to the AMF partner (Bever *et al*., 2009) that is perceived as being the most beneficial, whereas in other examples the hosts may invest large quantities of C for apparently little return (Walder *et al*., 2012). Biotic factors, such as the identity of the fungus and plant, and abiotic factors, such as nutrient availability (Johnson *et al*., 2015) and atmospheric CO_2_ concentrations (Field *et al*., 2012), may play important determining roles in mycorrhizal C‐for‐nutrient exchange. However, such factors are dynamic, often causing measurable changes in the stoichiometry of C for nutrient exchange between symbionts (Johnson *et al*., 1997).

Plants rarely associate with beneficial microorganisms alone; instead, they typically engage in concurrent interactions across a wide range of biotrophic symbionts, ranging from mutualists, including AMF, to parasites and pathogens, such as plant‐parasitic nematodes (PPNs). Soil‐dwelling PPNs evolved shortly after AMF (420 million years ago; Baldwin *et al*., 2004) and parasitize a wide range of economically important crops, causing > $80 billion per year in yield losses around the world (Nicol *et al*., 2011). Like AMF, the potato cyst nematode (PCN; *Globodera pallida*) occurs across all major potato‐growing regions and is an obligate biotroph on solanaceous plants (Moens *et al*., 2018). PCN can remain dormant in the soil as cysts, which each contain *c*. 400 eggs, for decades before hatching (Turner & Rowe, 2006). Upon hatching, the motile juvenile penetrates the host root tissue and migrates towards the vascular cylinder, where it selects a single cell to modify into a highly metabolically active feeding site, known as a syncytium. Syncytia acquire C and nutrients from the phloem in excess of surrounding tissues (Bockenhoff *et al*., 1996; Hofmann *et al*., 2009), supporting the development and subsequent egg laying of the next generation of PCN (Lilley *et al*., 2005).

Given that concurrent, biotrophic symbionts rely on their living plant host for a finite supply of resources, it stands to reason that competition between symbionts should be expected. Previous investigations into tripartite plant–aphid–AMF interactions have shown that aboveground aphid herbivory of host plants inhibits allocation of plant‐fixed C to AMF but that AMF‐mediated soil nutrient assimilation is maintained (Charters *et al*., 2020). This suggests that the relative sink strengths of competing symbionts may determine plant C allocation patterns. Though the varied benefits conferred on host plants by arbuscular mycorrhizal fungi are relatively well studied (Thirkell *et al*., 2017; Chen *et al*., 2018), the impact of pathogenic root symbionts on this near‐universal interaction has been almost entirely overlooked, representing a major knowledge gap. It is possible that host plants can discriminate between symbionts, preferentially allocating C resources to symbionts where a ‘benefit’ of association is perceived (Kiers *et al*., 2003; Bever *et al*., 2009). In this case, an increased supply of soil nutrients via AMF might be rewarded with allocation of recent photosynthates. Alternatively, it is possible that, because of their close proximity to one another within the root system, plants are unable to differentiate between competing root symbionts and that C allocation to roots is subsequently limited.

The application of AMF is regularly proposed to mitigate crop losses by enhancing plant tolerance and/or resistance to PPNs; however, data are often equivocal and vary considerably for fungal, nematode, and crop species, as well as for abiotic parameters (Schouteden *et al*., 2015). AMF may prime plant defences, which can help to prepare the host and strengthen their response upon PPN arrival (Vos *et al*., 2013). This synergistic effect increases the importance of AMF interactions within nature (Cameron *et al*., 2013). A crucial aspect of tolerance is the nutrient status of the host and the allocation of host resources. The impact of one obligate biotroph (i.e. AMF) on another (i.e. PCN), might indicate that there are systemic effects induced by mycorrhizal fungi, with a shift in host resource allocation a probable factor. The distribution of plant‐fixed C resources between plants and their competing root symbionts represents a critical gap in our understanding of belowground C dynamics (Bell *et al*., 2021).

We address this knowledge gap by examining the impact of belowground competition by PCN (*G. pallida*) for host resources on mycorrhizal C‐for‐nutrient exchange between potato (*Solanum tuberosum* cv Désirée) and the near‐ubiquitous AMF species *Rhizophagus irregularis*. We also investigate the impact of host symbiosis with AMF on the nutritional and fitness outcomes for PCN co‐symbionts.

## Materials and Methods

### Application and culture of arbuscular mycorrhizal fungi and potato cyst nematodes on potato plants

Cysts of the white PCN, *G. pallida* (population Lindley), were extracted from infected sand/loam using Fenwick's (1940) method. Cysts were subsequently stored dry at 4°C until further use. Potato tubers (*S. tuberosum* cv Désirée) with one chit present were planted in 21 cm diameter pots containing sterilized sand : topsoil (50 : 50) and either 30 g of a commercially available inoculum of the AMF *R. irregularis* (PlantWorks Ltd, Sittingbourne, UK) (AMF‐only treatment), 50 PCN cysts and 30 g of the AMF inoculum (AMF + PCN treatment), or 50 PCN cysts and 30 g of sterilized AMF inoculum to control for nutrients in the carrier substrate (PCN‐only treatment). Plants with no PCN plus 30 g of sterilized AMF inoculum were also set up as additional controls to assess the impact of AMF‐only, PCN‐only, or concurrent AMF and PCN treatments on the potato host (Fig. 2). Ten replicates were prepared for each treatment. Plants were grown in a randomized layout in a containment glasshouse with a controlled environment (18°C : 22°C, 16 h : 16 h day length) and watered every other day. Five weeks after planting, Chl content and *F*_V_/*F*_M_ were quantified with handheld devices (CL‐01; Hansatech, Kings Lynn, UK; OS30P; Opti‐Sciences Inc., Hudson, NH, USA) to measure plant photosynthetic capability and efficiency as indicators of host stress (Cessna *et al*., 2010).

### Experimental design

Based on established methods (Field *et al*., 2012), three windowed polyvinyl chloride cores of 20 mm diameter and 130 mm length (PlastOk Ltd, Birkenhead, UK) lined with 35 µm pore nylon mesh windows affixed to the sides and base of the cores using Tensol 12 acrylic adhesive (Bostik Ltd, Stafford, UK) were inserted into each pot. The mesh covering the windows was fine enough to exclude potato roots while permitting access to fungal hyphae (Johnson *et al*., 2001). A 1 mm diameter silicone capillary tube (Smiths Medical Ltd, Ashford, UK) perforated every 0.5 cm using a mounted needle was also fixed centrally in two of the three cores. This allowed for an even distribution of ^33^P and ^15^N aqueous solution through one core in each pot (see ^33^P and ^15^N isotope tracing) and water in the control core. At the time of planting, cores were filled with sterilized sand : loam to *c*. 90% of the core volume. The third core was filled with perlite and sealed with a gas‐tight Suba‐Seal rubber septum (Sigma‐Aldrich, Darmstadt, Germany) to enable nondestructive belowground gas sampling throughout the ^14^C labelling phase (‘see “Carbon‐14 labelling” in the Materials and Methods section’).

### Phosphorus‐33 and nitrogen‐15 tracing

At 5 wk, 150 µl of ^33^P‐orthophosphate (1.5 MBq; [^33^P]‐phosphoric acid; Hartmann Analytic, Braunschweig, Germany) and ^15^N ammonium chloride (1.5 mg ml^−1^; Sigma‐Aldrich) were supplied in an aqueous solution to one of the two mesh‐walled cores in each pot via the capillary tube. In half of the pots, the labelled cores were rotated every other day to sever the hyphal connection between core and plant roots (*n* = 6, ‘rotated’ cores). The nonlabelled cores in these pots were kept static to preserve the hyphal link. In the remaining half of the pots, the labelled cores were kept static, to preserve hyphal connections (*n* = 6, ‘static’ cores), and the nonlabelled cores were rotated.

### Carbon‐14 labelling

Two weeks after the application of the liquid isotope tracers, the aboveground tissue of plants was enclosed within airtight labelling chambers (Polybags Ltd, London, UK). At the beginning of the photoperiod, ^14^CO_2_ was liberated into the chamber by adding 2 ml 10% lactic acid to 110 µl ^14^C‐sodium bicarbonate (1 MBq; Hartmann Analytic). A 1 ml sample of labelled headspace gas was immediately obtained using a hypodermic syringe and every 90 min thereafter to monitor the drawdown of ^14^CO_2_ by plants. Belowground gas samples were taken via the perlite‐filled core immediately following the liberation of ^14^C and every 90 min thereafter, measuring respiration and flux of ^14^CO_2_ by the AMF. Above and belowground gas samples were injected into separate gas‐evacuated 20 ml scintillation vials containing equal volumes of the C‐trapping cocktail Carbo‐Sorb and liquid scintillant Permafluor (PerkinElmer, Beaconsfield, UK).

### Harvesting and preparation of material

Plants were left *in situ* until a maximum of ^14^C flux was detected belowground (24 h post‐labelling), at which point 4 ml of 2 M potassium hydroxide (KOH) was injected into receptacles inside each airtight chamber to capture the remaining ^14^CO_2_ gas. Soil cores were then removed from the pots and plant material was separated into shoots, roots and tubers. Soil was separated into soil from the rotated and static cores as well as bulk soil from the pot. Nematode cysts were extracted by vigorously shaking the roots/soil and using Fenwick's (1940) method. A 200 g subsample of roots/soil was used for this method, with the resulting data then scaled up by pot mass to represent the entire PCN population on the host. All individual components were weighed fresh.

Roots were cleaned with tap water and a subsample taken for quantification of arbuscular mycorrhizal (AM) root length colonization and stored in 50% ethanol (v/v) at 4°C until further use. A 10 g sample of bulk soil was also stored at −20°C for quantification of AM hyphal lengths. All remaining components were stored at −20°C until they were freeze‐dried (CoolSafe 55‐4; LaboGene, Allerød, Denmark). DW measurements of each component were taken, before being analysed for P, ^33^P, ^15^N, and ^14^C.

### Plant and mycorrhizal‐acquired phosphorus, phosphorus‐33, and nitrogen‐15

To quantify ^33^P and ^15^N activity in plant, soil, and nematodes, respective samples were digested in concentrated acid and a diluted subsample underwent liquid scintillation (Packard Tri‐Carb 3100TR; PerkinElmer). Approximately 100–200 mg of the homogenized freeze‐dried samples was digested in duplicate in 2 ml concentrated sulphuric acid at 365°C for 15 min. Hydrogen peroxide (100 ml) was added to each cooled sample and returned to the digest block briefly until the solution cleared (Grant BT5D; Grant Instruments (Cambridge) Ltd, St Ives, UK). Cleared digest solutions were then diluted to 10 ml with distilled water (dH_2_O). Sample radioactivity was quantified through liquid scintillation (Tri‐Carb 3100TR; PerkinElmer). Then, 2 ml of each diluted digest solution was added to 10 ml of the liquid scintillant (Emulsify‐safe; PerkinElmer), and previously published equations were then applied to calculate the quantity of ^33^P (Cameron *et al*., 2007) in each component of each experimental pot. The mean ^33^P content of plants that did not have hyphal access to the isotopes (‘rotated’ core pots) was subtracted from the ^33^P content in each plant that did have hyphal access to the isotopes (‘static’ core pots), to determine total fungal‐acquired ^33^P and control for the movement of isotopes out of the cores by diffusion or alternative microbial nutrient cycling processes.

Approximately 2–6 mg of each sample was analysed for ^15^N content by continuous flow infrared mass spectrometry (PDZ 2020 IRMS; Sercon Ltd, Crewe, UK). The amount of ^15^N transferred from the fungus to the plant and the nematodes was calculated using equations published previously (Field *et al*., 2016).

Total P content of plant material was determined using an adapted method (Murphy & Riley, 1962). We then added *c*. 0.15 ml and *c*. 0.2 ml of sulphuric acid digest solutions to separate cuvettes with 0.5 ml ammonium molybdate, 0.2 ml of 0.1 M l‐ascorbic acid, and 0.2 ml 3.44 M sodium hydroxide. Solutions were made up to 3.8 ml with dH_2_O. Sample optical density was recorded after 45 min at 822 nm using a spectrophotometer (Jenway 6300; Cole‐Parmer, Stone, UK). A 10 mg ml^−1^ standard P solution was used to produce a standard curve against which total sample P (milligrams) was calculated.

### Plant carbon transfer to the arbuscular mycorrhizal fungus and assimilation by nematodes

To quantify ^14^C activity, samples underwent oxidation (Model 307 Packard Sample Oxidiser; Isotech) followed by liquid scintillation (Packard Tri‐Carb 3100TR; PerkinElmer). Approximately 10–100 mg of each freeze‐dried sample was weighed in triplicate into Combusto‐cones (PerkinElmer). ^14^C within each material was measured following sample oxidation (Model 307 Packard Sample Oxidiser; Isotech) whereby released CO_2_ gets trapped in 10 ml of the C‐trapping cocktail Carbo‐Sorb and mixed with 10 ml of the liquid scintillant Permafluor (Meridian Biotechnologies Ltd, Tadworth, UK). Radioactivity within samples was then quantified through liquid scintillation counting (Packard Tri‐carbon 3100 TR; Isotech). Using established equations (Cameron *et al*., 2008), the total C fixed by plants (i.e. ^12^CO_2_ and ^14^CO_2_) and transferred to their symbionts was calculated. The difference in total C between the static and rotated cores represents the total C transferred from plant to AMF.

### Fungal colonization of roots and bulk substrates

Root samples were cleared in 10% KOH (w/v) in a 90°C water bath for 20 min and AM fungal structures stained with ink and vinegar (5% Pelikan Brilliant Black, 5% acetic acid, 90% dH_2_O) (Vierheilig *et al*., 1998) in a 90°C water bath for 15 min. Roots were de‐stained in 1% acetic acid and mounted on microscope slides using polyvinyl lacto‐glycerol (16.6 g polyvinyl alcohol powder, 10 ml glycerol, 100 ml lactic acid, 100 ml dH_2_O). Assessments of percentage root length colonization were made using the magnified intersection methodology (minimum of 150 intersects per pot, ×200 magnification) (McGonigle *et al*., 1990).

Mycorrhizal hyphae were extracted from 5 g of bulk soil in 500 ml water, from which 10 ml was filtered through two 0.45 mm membrane filters (Whatman plc, Maidstone, UK) and stained with ink and vinegar. Filter papers were mounted on microscope slides using polyvinyl lacto‐glycerol and then oven‐dried at 65°C overnight. AM hyphal lengths per pot were calculated using the gridline‐intersection methodology (50 fields of view per filter paper, ×200 magnification) (Tennant, 1975).

After harvest, the soil was well mixed for spore extraction (Brundrett *et al*., 1994), and then 25 g soil was decanted through 750, 250, and 40 µm sieves. Sieves were washed until the water ran clear. The contents of the 40 µm sieve were collected into 50% sucrose and centrifuged at 2000 ***g*** for 2 min. The supernatant, containing the spores, was then washed on the 40 µm sieve to remove the sucrose. The contents were then washed into a 50 ml polypropylene tube for counting.

### Quantification of potato cyst nematode infection

PCN cysts were extracted from the soil at the end of the experiment by vigorously shaking the roots/soil and using Fenwick's (1940) method. These data were used alongside root DW data to provide cysts per gram of root. Ten recovered cysts were opened by crushing under a brass plate, and the number of eggs within each cyst was counted and expressed as eggs per cyst.

### Data analysis

Statistics were carried out using OriginPro (OriginLab Corp., 2021). Data were tested for normality using the Shapiro–Wilk test. Data were analysed by one‐way ANOVA. Upon *P* < 0.05, *post hoc* Student–Newman–Keuls (SNK) tests were run to identify statistical differences between the treatments. Data with two variables were compared by two‐sample *t*‐tests.

## Results

### Arbuscular mycorrhizal fungi colonization increases the potato cyst nematode population on the root

**Fig. 1:**
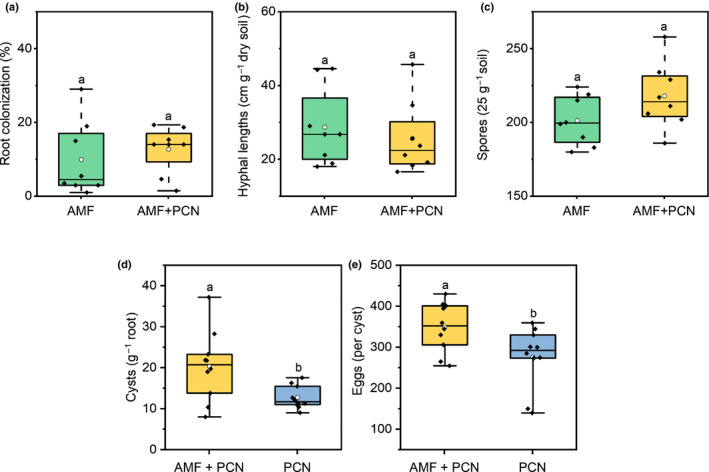
The fitness of both arbuscular mycorrhizal fungi (AMF) and potato cyst nematodes (PCN) on potato roots inoculated with AMF only (green), dual co‐inoculation (yellow), and PCN only (blue). (a) Root colonization, (b) hyphal lengths, and (c) spore content of the soil as markers of AMF fitness (*n* = 8). (d) PCN concentration on the root system and (e) the number of PCN eggs within each cyst (*n* = 10). Boxplots extend from the first to the third quartiles, with the middle bold line representing the median value, the white circle representing the mean, and whiskers extending to the minimum and maximum data points. Different letters denote significance (*P* < 0.05, two‐sample *t‐*test).

The percentage root colonization by AMF and the hyphal and spore content of the surrounding soil were unaffected by concurrent PCN infection on the roots (Fig. 1a–c). Conversely, the presence of AMF increased the number of PCN that invaded the root and developed into cysts (Fig. 1d; *P* < 0.05, two‐sample *t‐*test). PCN that invaded the hosts produced a greater number of eggs when the roots were also colonized by AMF (Fig. 1e; *P* < 0.05, two‐sample *t‐*test).

### Mycorrhizal fungal colonization enhances plant growth and ameliorates detrimental effects of potato cyst nematodes despite increasing potato cyst nematode infection

**Fig. 2:**
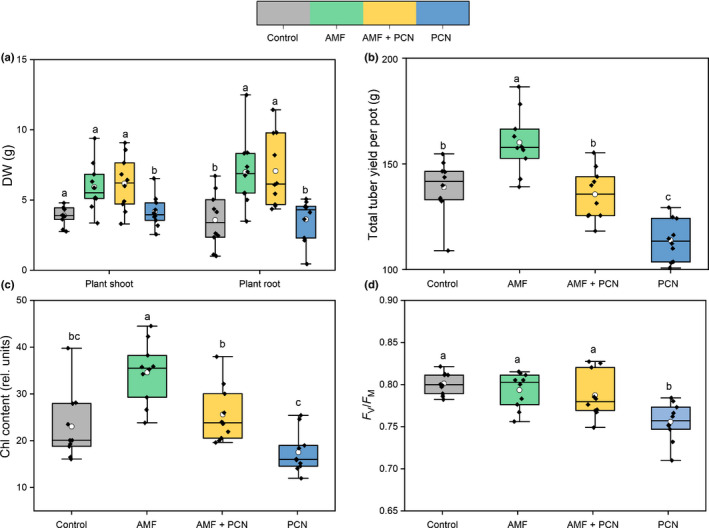
The impact of arbuscular mycorrhizal fungi (AMF) only (green), potato cyst nematodes (PCN) only (blue), or concurrent AMF and PCN (yellow) on the potato host. (a) DW of shoot and root tissue. (b) Total tuber yield. (c) Chl content at week 7, prior to radiolabelling. (d) Photosynthetic efficiency *F*_V_/*F*_M_ at week 7, prior to radiolabelling. All plots represent 10 biological replicates with either control, AMF, or PCN treatments. Boxplots extend from the first to the third quartiles, with the middle bold line representing the median value, the white circle representing the mean, and whiskers extending to the minimum and maximum data points. Different letters denote significance where *P* < 0.05 (one‐way ANOVA, Student–Newman–Keuls).

Potato plants colonized by AMF, even in the presence of PCN, were larger both above and belowground compared with asymbiotic control plants (Fig. 2a; *P* < 0.05, one‐way ANOVA, SNK). Infection only with PCN did not affect shoots and root biomass; however, tuber yields were reduced (Fig. 2b; *P* < 0.05, one‐way ANOVA, SNK). Tuber biomass (yield) was increased from plants colonized solely by AMF, whereas dual AMF and PCN colonized host yields were not different to those of asymbiotic control plants (Fig. 2b), despite a higher nematode population (Fig. 1d,e).

Chl content was greater in plants colonized only by AMF and was reduced in PCN‐only plants compared with asymbiotic control plants (Fig. 2c; *P *< 0.05, one‐way ANOVA, SNK). Concurrent AMF and PCN colonization offset these effects, with Chl content in dual‐colonized plants being the same as in asymbiotic control plants. *F*_v_/*F*_m_ (photosynthetic efficiency) was reduced in PCN‐only host plants (Fig. 2d; *P* < 0.05, one‐way ANOVA, SNK). Although *F*_v_/*F*_m_ was not increased in AMF‐only hosts relative to controls, AMF colonization appeared to offset PCN‐induced reduction to *F*_v_/*F*_m_ when both symbionts were simultaneously present (*P* < 0.05, one‐way ANOVA, SNK).

### Phosphorus‐33 transfer from fungus to plant is modulated by the addition of potato cyst nematodes

**Fig. 3:**
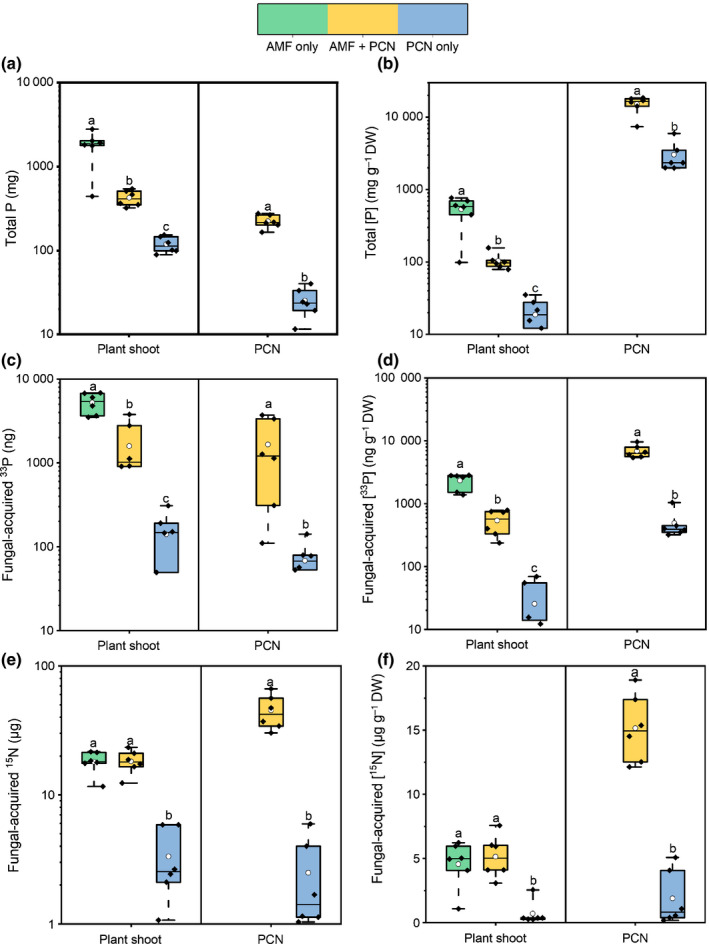
Phosphorus (P) and nitrogen (N) assimilation and distribution in plants with arbuscular mycorrhizal fungi (AMF) only (green), potato cyst nematodes (PCN) only (blue), or concurrent AMF and PCN symbionts (yellow). (a) Shoot and PCN P content. (b) Shoot and PCN P concentration. (c) AMF‐acquired shoot and PCN ^33^P content. (d) AMF‐acquired shoot and PCN ^33^P concentration. (e) AMF‐acquired shoot and PCN ^15^N content. (f) AMF‐acquired shoot and PCN ^15^N concentration. PCN data were determined from nematode females collected by Fenwick's (1940) method. The data from this sample were then scaled up to represent the total PCN population on the plant. Boxplots represent six biological replicates and extend from the first to the third quartiles, with the middle bold line representing the median value, the white circle representing the mean, and whiskers extending to the minimum and maximum data points. Different letters denote significance within panel boxes (*P* < 0.05, one‐way ANOVA, Student–Newman–Keuls).

Total shoot P was greater in plants colonized by AMF, compared with PCN‐only plants (Fig. 3a,b; *P* < 0.01, one‐way ANOVA, SNK). Total shoot P represents P obtained from the fungus and the roots; therefore, we used ^33^P‐labelled orthophosphate to trace AMF‐acquired resources. AMF‐acquired ^33^P was approximately three‐fold lower in the shoots of plants that also had PCN symbionts (Fig. 3c,d; *P* < 0.01, one‐way ANOVA, SNK). The reduced amount observed in hosts with both AMF and PCN was still much higher than in plants without AMF present, indicating that the host still benefits from P assimilation via AMF, even when PCN are also present (*P* < 0.01; one‐way ANOVA, SNK). ^15^N was applied alongside ^33^P to track the transfer of AMF‐acquired ^15^N to the host. In plants with only AMF symbionts present, AMF transferred ^15^N to host tissue (Fig. 3e). Concurrent infection with PCN did not alter the amount of ^15^N transferred to host shoots (*P* > 0.05, two‐sample *t*‐test).

^33^P and ^15^N were detected in the feeding PCN in greater concentrations when AMF were present (Fig. 3c,f; *P* < 0.01, one‐way ANOVA, SNK). The relatively high concentrations of AMF‐derived ^33^P and ^15^N present in PCN suggest that PCN are potentially a stronger sink for these nutrients than the host plant per unit of biomass (Fig. 3d,f).

### Infection by potato cyst nematodes restricts carbon allocation to arbuscular mycorrhizal fungi

**Fig. 4:**
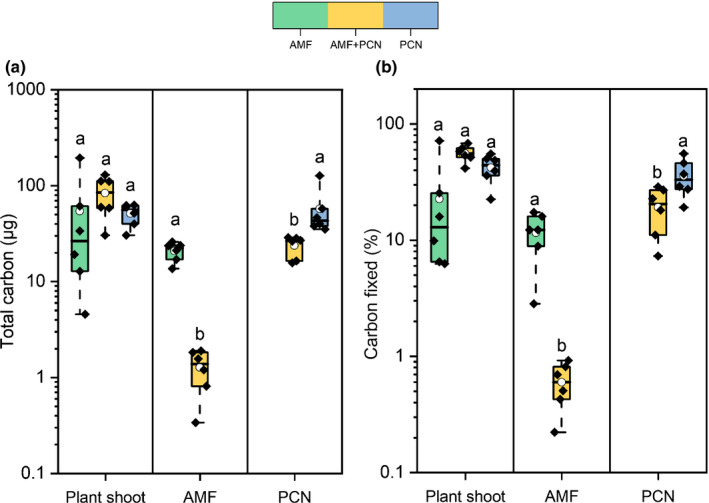
Plant carbon (C) allocation to the shoots, extraradical arbuscular mycorrhizal fungi (AMF) hyphae, and potato cyst nematodes (PCN) in plants colonized by only AMF (green), PCN (blue), or dual colonizations of AMF and PCN (yellow). (a) Total C fixed by plants during the labelling period and allocated to plant material, AMF, or PCN. (b) Percentage of fixed C allocated to different symbionts or plant material. Boxplots represent six biological replicates and extend from the first to the third quartiles, with the middle bold line representing the median value, the white circle representing the mean, and whiskers extending to the minimum and maximum data points. Different letters denote significance within panel boxes (*P* < 0.05, one‐way ANOVA, Student–Newman–Keuls).

Of the recently fixed photosynthates, *c*. 12% were transferred to AMF and 35% to PCN in plant hosts with a single symbiont (Fig. 4b). In plants concurrently hosting AMF and PCN, transfer of plant*‐*fixed C to the AMF was reduced 20‐fold to 0.6% (Fig. 4b; *P* < 0.001, one‐way ANOVA, SNK). In these same hosts, allocation of fixed C to the PCN was reduced to 20%.

PCN‐infected hosts distributed significantly less C belowground to their root and tuber components compared with AMF‐only hosts (Supporting Information Fig. S1; *P* < 0.05; one‐way ANOVA, SNK). Additionally, the percentage allocation of plant‐fixed C to aboveground shoot tissues was increased when PCN were present (combined PCN‐sonly and AMF + PCN datasets), compared with hosts without PCN (AMF‐only; Fig. 4a; *P* < 0.01, two‐sample *t*‐test).

**Fig. 5:**
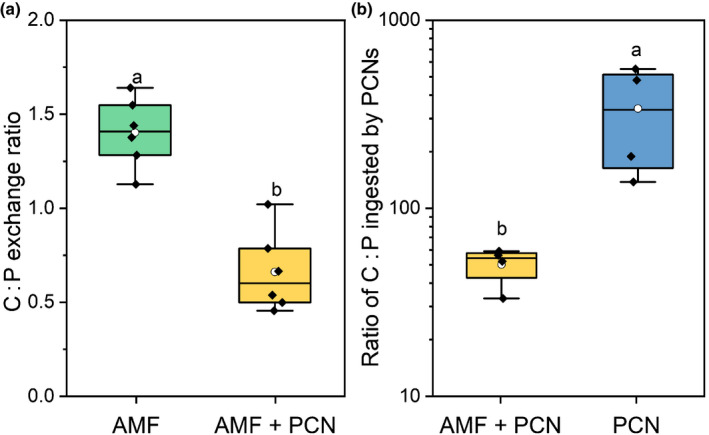
The ratio of plant fixed carbon (C) and arbuscular mycorrhizal fungi (AMF)‐acquired phosphorus (P) exchanged by the host and ingested by potato cyst nematodes (PCN). Data points indicate (a) the ratio of plant‐fixed C exchanged with fungal‐acquired P in the host plants and (b) the ratio of these components in feeding PCN. Data are from potato plant treatments under AMF only (green), AMF and PCN (yellow), and PCN only (blue). Boxplots represent six biological replicates and extend from the first to the third quartiles, with the middle bold line representing the median value, the white circle representing the mean, and whiskers extending to the minimum and maximum data points. Different letters denote significance (*P* < 0.01, two‐sample *t‐*test).

The exchange ratio of C for P between host and AMF can infer the relative costs for transferring each resource. When they are the only symbiont present, AMF receive *c*. 1.4 units of C for each unit of ^33^P transferred to the host; however, when PCN were also present then AMF receive *c*. 0.65 units of C for each unit of ^33^P transferred to the host (Fig. 5a; *P* < 0.01, two‐sample *t‐*test). The ratio of plant‐fixed C and fungal‐acquired ^33^P ingested by the feeding PCN can also be quantified. PCN ingested *c*. 300 times more C than P when infecting a non‐AMF‐colonized host (Fig. 5b). However, when AMF are also present, and greater P was available, then the C : P was reduced to *c*. 50 times more C than P (*P* < 0.05, two‐sample *t‐*test).

## Discussion

### Arbuscular mycorrhizal fungi colonization of host plants resulted in increased potato cyst nematode abundance and fitness

The presence of PCN did not affect the root colonization, spore density, or extraradical hyphal lengths of AMF in our experiments (Fig. 1). Previous research has similarly shown plant root colonization by AMF to be largely unaffected by migratory PPNs (Elsen *et al*., 2003; Koffi *et al*., 2013; van der Veken *et al*., 2021), but this contrasts with other reports that found it to be negatively impacted by sedentary PPNs, including PCN (Deliopoulos *et al*., 2008; dos Anjos *et al*., 2010; Alguacil *et al*., 2011; Alban *et al*., 2013). The variation between our observations and previous studies may be due to the varying levels of tolerance/resistance of different potato varieties towards PCN and the intraspecific diversity between populations of each symbiont, as species identity is known to play a large role in biotic interactions (Veresoglou & Rillig, 2012; Salvioli & Bonfante, 2013; Gough *et al*., 2020). Additionally, the population density of the symbiont can have a large impact on the symbioses (Gartner *et al*., 2021) yet remains to be investigated within this system.

The PCN in our experiments reproduced in host roots at higher rates on plants colonized by AMF than on asymbiotic plants (Fig. 1). This finding seems, at first, counterintuitive, given that colonization by AMF is widely reported to induce, or prime, plant defence responses (Cameron *et al*., 2013; Schouteden *et al*., 2015), with previous research having shown that AMF colonization can exert a biocontrol effect, reducing the incidence/severity of pests and/or disease (Schouteden *et al*., 2015; Olowe *et al*., 2018). Positive effects of AMF on PPN populations have been reported previously, but this is contradictory to the majority of studies that report negative biocontrol effects (Schouteden *et al*., 2015; Frew *et al*., 2018; Gough *et al*., 2020, 2021). The increase in abundance and fitness of the PCN on plants colonized by AMF compared with asymbiotic plants in our experiments (Fig. 1) may be due to enhanced host plant nutritional value via the mutualistic actions of their AMF symbionts. As obligate biotrophs, PCN rely on their host to fulfil their entire nutritional requirements, with often devastating impacts for their host plants in terms of fitness and/or yield (Moens *et al*., 2018). However, the relative demand of PCN for specific nutrients remains largely unknown.

AMF are well‐documented to deliver both P and N to their host plant (Smith & Read, 2008) (Fig. 3), and there is evidence that such transfers are highly context dependent (Johnson *et al*., 1997). Transfer of N and P to plants by AMF is known to be affected by environmental changes (Field *et al*., 2012) and nutrient availability in the soil (Johnson *et al*., 2015), and also biotic interactions such as competition from neighbouring plants (Walder *et al*., 2012) or aphid herbivory (Charters *et al*., 2020). In our experiments, colonization by PCN caused a reduction in both the total amount of P in aboveground plant tissues and mycorrhizal‐acquired ^33^P tracers when compared with plants that were colonized by AMF alone (Fig. 3). The reduction in P in the shoots may be a direct consequence of preferential uptake of P by PCN, thereby removing it from the root tissues before it can be moved to the shoot. The decrease in total P under AMF + PCN treatments also indicates that, when there is a reduced amount of mycorrhizal‐acquired P entering the roots, the host does not compensate by acquiring additional amounts of P through its own mechanisms. The transfer of mycorrhizal‐acquired ^15^N was unaffected by the presence of PCN. This is consistent with previous reports (Charters *et al*., 2020) where the presence of aphids did not impact the transfer of AMF‐acquired nutrients to the host.

In our experiments, AMF‐acquired nutrients (^15^N and ^33^P) were assimilated by PCN, contributing towards their total nutrient capture from their host plants (Fig. 3). AMF‐acquired ^33^P was assimilated into PCN in co‐colonization treatments in amounts comparable to that assimilated into plant shoots. However, given their much lower biomass, the concentration of AMF‐acquired ^33^P in PCN was much higher than it was for host plant shoots (Fig. 3). This suggests that PCN represent much greater sink strength for soil nutrients than the host plant does, potentially explaining why co‐colonized plants assimilated less AMF‐acquired ^33^P in shoots than plants that were not parasitized by PCN. Similarly for N, PCN assimilated more AMF‐acquired ^15^N tracer than host plant shoots did, suggesting that they represent a greater sink strength for mycorrhizal N than the host plant does. This is the first instance where the acquisition of AMF‐derived nutrients by nematodes has been observed, indicating that these nutrients may also be available to other host symbionts. It is possible that stoichiometric differences in tissue C : N : P ratios of different symbionts drive differences in sink strengths (Allen *et al*., 2003; Zhang *et al*., 2021). Indeed, different species of PPNs can induce varying strength sinks (Carneiro *et al*., 1999), suggesting a complex scenario depending on which pest(s) is(are) present.

Taken together, our findings support the hypothesis that increased abundance and fitness of PCN on plants associated with AMF compared with asymbiotic plants is, at least in part, due to an enhanced host plant nutrient supply. The enhanced nutrient status of plants when colonized by AMF appears to lead to greater P and N acquisition by PCN during feeding, leading to a greater number of eggs being produced. This is supported by previous studies that have shown that plants with greater nutritional value support higher densities of PPNs (Pettigrew *et al*., 2005). Rather than mycorrhizal‐induced resistance (MIR), which induces plant defence responses to defeat incoming pathogens (Cameron *et al*., 2013; Schouteden *et al*., 2015), the mycorrhizal‐induced tolerance (MIT) outlined here may enable a susceptible host to continue to produce high yields despite the continued presence and success of parasites. A recent study in mung bean infected with a migratory PPN alongside AMF reported similar MIT responses (Gough *et al*., 2021). The enhanced photosynthetic activity, biomass, and yields of PCN‐infected mycorrhizal plants observed here indicate that MIT confers numerous positive physiological benefits on the host plant. How strongly MIT performs in various scenarios, such as at higher PCN densities and in a field environment with potentially numerous symbionts, remains to be investigated. In systems such as ours, where the host is entirely susceptible to the pest, MIT is perhaps of greater importance than MIR.

### Potato cyst nematode caused reduction in carbon allocated to AMF symbionts whilst nitrogen and phosphorus supply was maintained

In exchange for fungal‐acquired N and P, AMF are supplied with organic C from host plants as lipids (Luginbuehl *et al*., 2017) and/or hexose sugars (Helber *et al*., 2011). Given that mycorrhizal‐acquired P is reduced in host plants co‐colonized by PCN, it might be expected that plant C supply to AMF partners also diminishes in line with a ‘reciprocal rewards’ mechanism (Kiers *et al*., 2011). When PCN were present in our experiments, the amount of C allocated to AMF by the host plant was dramatically reduced (Fig. 4), falling from 12% of the total amount of C fixed in plants without PCN to 0.6% when PCN co‐colonized the root system, representing a 20‐fold reduction. Despite there being very little plant C transferred to AMF in PCN‐colonized plants, AMF continued to supply their host plant with ^33^P (Fig. 3c), and ^15^N supply was unaffected (Fig. 3e). It is possible that the amount of C received by AMF from PCN‐colonized plants represented the minimum required to maintain AMF symbionts in our experimental systems. Given that our experiments comprised single plants, alternative sources of organic C were not available, and this may have forced AMF to form apparently unfavourable symbioses with hosts that do not readily transfer C resources.

Aphid herbivory has been shown to similarly reduce host plant C transfer to mycorrhizas whilst transfer of fungal‐acquired nutrients to the plant is maintained, potentially as a result of aphids feeding directly from the C‐fixing tissue, thereby locally disrupting C fixation and flow to the host roots (Charters *et al*., 2020). However, this is unlikely to be the cause of reduced C supply to the AMF in our experiment, given that, unlike aphids, PCN do not feed directly from the host phloem or C‐fixing tissue but instead feed from specially modified cells in the roots that can be in close vicinity to cells colonized by AMF. Given the deleterious outcomes for the host plant of PCN infection, there may be a PCN‐controlled, systemic redirection of C from the foliar tissue towards the PCN feeding sites rather than towards the AMF. Despite the apparent redirection of resources towards PCN, there is also an overall reduction in plant C allocation belowground, suggesting that plant C is withheld/translocated aboveground and potentially invested in defence responses. This indicates that the presence of PCN triggers the host plant to divert C resources away from the sites of C loss. Suppression of host photosynthesis has been previously reported for hosts infected with PPNs (Blouin *et al*., 2005), and it is possible that this also contributed to the reduction of C allocated to belowground plant parts and symbionts. Consistent with this hypothesis, AMF‐only plants had greater Chl content in their leaves (Fig. 2c), allowing greater C fixing and movement to belowground tissue, potentially regulated by relative sink strengths (Walder & Van Der Heijden, 2015). Although PCN infection did not affect root colonization by AMF in our experiment (Fig. 1), PCN potentially impose a greater C sink strength than AMF within the same root system, meaning more host C was drawn towards the PCN. PPNs have been shown to exert a negative impact on other root symbiotic microorganisms that rely on plant C (Elhady *et al*., 2020). Although the mechanism underpinning this is unclear, it seems that reduced C allocation to symbionts due to the greater sink strength of the parasites could play an important role.

AMF rely on their host plant to fulfil their entire C requirements (Smith & Read, 2008). In nature, neighbouring plants within communities are typically interconnected by common mycelial networks (CMNs) of shared AMF hyphae (Alaux *et al*., 2021). AMF‐acquired nutrients and plant‐fixed C are distributed throughout these networks (Robinson, 1999; Bücking *et al*., 2016), reducing the reliance of AMF on individual plant hosts for plant C. In this way, plant communities may not only share the benefits of AMF symbionts but also share, and thereby mitigate, the cost of symbiosis and the burden of pests through mobilization and redistribution of resources through the network (Bücking *et al*., 2016). In this way, plants infected with PCN may still benefit from AMF associations in terms of nutrient gains, but the C demand of AMF may be fulfilled by neighbouring plants from across the network. This strategy, together with the ability of the CMNs to transmit plant defence signals to neighbouring plants (Babikova *et al*., 2013), could help enhance resilience against pests and pathogens in plant communities in both natural and agroecosystems (Alaux *et al*., 2021). The host also may interact with multiple AMF taxa simultaneously (Chen *et al*., 2017), which may impact plant defence responses and C : N exchange. These interactions may introduce a wider range and/or increased quantity of nutrients to the host, but it may increase the symbiont burden on the host and further reduce the allocation of host C to each AMF.

Our results demonstrate that AMF‐acquired resources may not solely benefit the host plant but may also enhance the fitness of parasites while simultaneously lending tolerance to the host plant against an increasing parasite burden. These simultaneous nutritional and nonnutritional benefits conferred by AMF to hosts and their parasites are seldom considered in tandem in plant community ecology and are likely to play an important role in both plant and soil community structure. This may be a critical oversight, particularly in the consideration of C and nutrient flows in plant and soil communities, and more broadly across terrestrial ecosystems. Further research is now required to unpick the regulatory mechanisms underpinning resource exchanges in concurrent, multi‐symbiont scenarios.

## Author contributions

All authors designed the research plans; CAB and EM performed the experimental work under PEU and KJF supervision; CAB analysed the data and wrote the manuscript; all authors read and approved the final version.

## Supporting information


**Fig. S1** Plant carbon allocated to the roots and tubers in plants inoculated with AMF‐only, PCN‐only or concurrent AMF and PCN.
Please note: Wiley Blackwell are not responsible for the content or functionality of any Supporting Information supplied by the authors. Any queries (other than missing material) should be directed to the *New Phytologist* Central Office.

## Data Availability

The data that support the findings of this study are available from the corresponding author upon reasonable request.
