# Supplementary material for: Disruption of carbon for nutrient exchange between potato and arbuscular mycorrhizal fungi enhanced cyst nematode fitness and host pest tolerance
Source: New Phytol. 2022 Feb 2;234(1):269–79. doi: 10.1111/nph.17958 (PMC9304131; doi:10.1111/nph.17958)
Supplement: Supplementary file 1 — Fig. S1 Plant carbon allocated to the roots and tubers in plants inoculated with AMF‐only, PCN‐only or concurrent AMF and PCN. Please note: Wiley Blackwell are not responsible for the content or functionality of any Supporting Information supplied by the authors. Any queries (other than missing material) should be directed to the New Phytologist Central Office. [file NPH-234-269-s001.pdf]

***New Phytologist* Supporting Information**

Article title: Disruption of carbon for nutrient exchange between potato and arbuscular mycorrhizal fungi enhances cyst nematode fitness and host pest tolerance

Authors: Christopher A. Bell[1*], Emily Magkourilou[1,2], P. E. Urwin[1] and Katie J. Field[2]

[1] School of Biology, Faculty of Biological Sciences, University of Leeds, Leeds, LS2 9JT [2] School of Biosciences, University of Sheffield, Sheffield, S10 2TN *Corresponding author: Christopher A. Bell, c.a.bell@leeds.ac.uk
Article acceptance date: 16 December 2021

The following Supporting Information is available for this article:

Fig. S1 Plant carbon allocated to the roots and tubers in plants inoculated with AMF-only (green), PCN-only (blue) or concurrent AMF and PCN (yellow). Boxplots represent six biological replicates and extend from the first to the third quartile with the middle bold line representing the median value, the white circle representing the mean and whiskers extending to the minimum and maximum data points. Different letters denote significance within panels ($P<0.05$; One-way ANOVA, SNK).

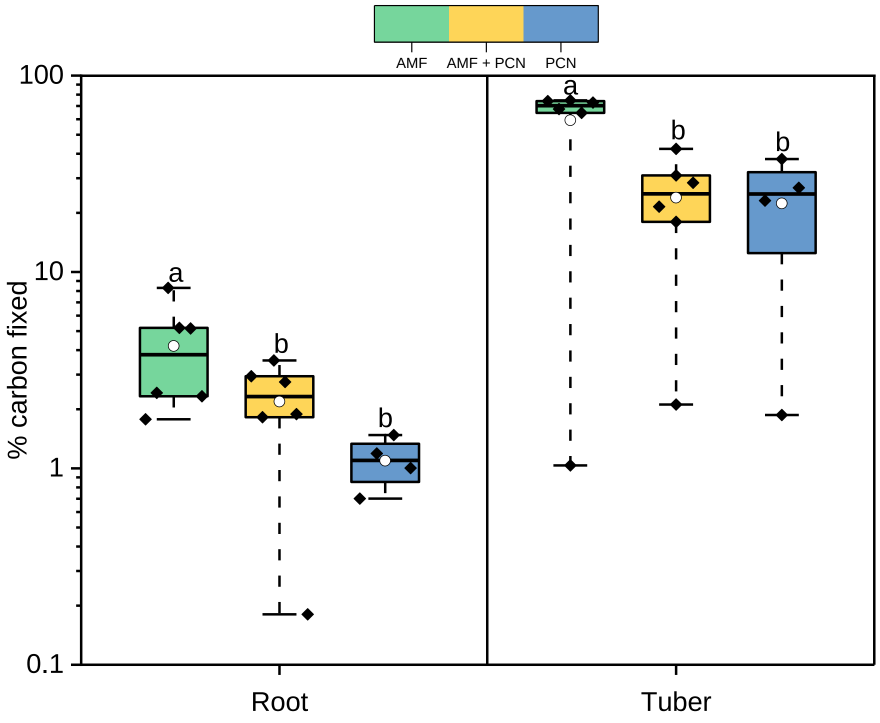

AMF
AMF + PCN
PCN
100
10
1
0.1
% carbon fixed
a
b
b
a
b
b
Root
Tuber
